# Dietary Silicon Deficiency Does Not Exacerbate Diet-Induced Fatty Lesions in Female ApoE Knockout Mice^1^,^2^,^3^

**DOI:** 10.3945/jn.114.206193

**Published:** 2015-05-13

**Authors:** Ravin Jugdaohsingh, Katharina Kessler, Barbara Messner, Martin Stoiber, Liliana D Pedro, Heinrich Schima, Günther Laufer, Jonathan J Powell, David Bernhard

**Affiliations:** 4 Medical Research Council (MRC) Human Nutrition Research, Elsie Widdowson Laboratory, Cambridge, United Kingdom;; 5 Cardiac Surgery Research Laboratories, Department of Surgery, and; 6 Center for Medical Physics and Biomedical Engineering, Medical University of Vienna, Vienna, Austria;; 7 Ludwig-Boltzmann-Cluster for Cardiovascular Research, Vienna, Austria; and; 8 Cardiac Surgery Research Laboratory Innsbruck, University Clinic for Cardiac Surgery, Medical University of Innsbruck, Innsbruck, Austria

**Keywords:** aortic lesions, aorta silicon content, apoE knockout mice, collagen and elastin contents, dietary silicon, monomethylsilanetriol, elastic structure and morphology, nutrition, serum lipids, tensile strength and elasticity

## Abstract

**Background:** Dietary silicon has been positively linked with vascular health and protection against atherosclerotic plaque formation, but the mechanism of action is unclear.

**Objectives:** We investigated the effect of dietary silicon on *1*) serum and aorta silicon concentrations, *2*) the development of aortic lesions and serum lipid concentrations, and *3*) the structural and biomechanic properties of the aorta.

**Methods:** Two studies, of the same design, were conducted to address the above objectives. Female mice, lacking the apolipoprotein E (*apoE*) gene, and therefore susceptible to atherosclerosis, were separated into 3 groups of 10–15 mice, each exposed to a high-fat diet (21% wt milk fat and 1.5% wt cholesterol) but with differing concentrations of dietary silicon, namely: silicon-deprived (−Si; <3-μg silicon/g feed), silicon-replete in feed (+Si-feed; 100-μg silicon/g feed), and silicon-replete in drinking water (+Si-water; 115-μg silicon/mL) for 15–19 wk. Silicon supplementation was in the form of sodium metasilicate (feed) or monomethylsilanetriol (drinking water).

**Results:** The serum silicon concentration in the −Si group was significantly lower than in the +Si-feed (by up to 78%; *P* < 0.003) and the +Si-water (by up to 84%; *P* < 0.006) groups. The aorta silicon concentration was also lower in the −Si group than in the +Si-feed group (by 65%; *P* = 0.025), but not compared with the +Si-water group. There were no differences in serum and aorta silicon concentrations between the silicon-replete groups. Body weights, tissue wet weights at necropsy, and structural, biomechanic, and morphologic properties of the aorta were not affected by dietary silicon; nor were the development of fatty lesions and serum lipid concentrations.

**Conclusions:** These findings suggest that dietary silicon has no effect on atherosclerosis development and vascular health in the apoE mouse model of diet-induced atherosclerosis, contrary to the reported findings in the cholesterol-fed rabbit model.

## Introduction

Silicon, a major trace element of the daily diet (1, 2), may be important for the normal health of bone and connective tissues (3–6). This includes vascular tissue, where dietary silicon has been positively linked with cardiovascular health (7–13). Schwarz (11) reported an inverse association between silicon concentrations in drinking water and the number of deaths from cardiovascular disease in Finland. He proposed that a lack of silicon in the diet and in drinking water may contribute to the etiology of the disease, whereas sufficiency or excess may inhibit disease development. A similar association was reported by Dawson et al. (14). Indeed, silicon administered intravenously or orally to cholesterol-fed rabbits greatly reduced, and almost negated, the formation of cholesterol plaques (8). Maehira et al. (12) reported that soluble silica significantly reduced systolic blood pressure in spontaneously hypertensive rats, suggesting that silicon may reduce hypertension. Buffoli et al. (13) reported in a mouse model of physiologic aging that silicon supplementation in drinking water can protect against age-related vascular aging. These findings suggest a potential role for dietary silicon in vascular health, although the mechanism of action is unclear.

High concentrations of silicon are found in normal healthy connective tissues, including the aorta, where it is associated with the elastin and collagen components of the aortic wall (8, 15), and concentrations decrease with aging and with the progression of atherosclerosis (8, 10). Histologic analysis of the aortic wall of cholesterol-fed rabbits with and without silicon treatment found that the elastic fibers of the internal elastic lamina seemed “undamaged and often thickened” in the silicon-supplemented groups compared with the control group (8). These findings led to the suggestion that silicon may be an important structural component of the aortic wall that protects against lipid permeability (8). A further proposal is that silicon supplementation decreases the concentrations of FAs that are negatively linked to atherosclerotic plaque formation in serum and aorta (7, 9, 16). However, the changes in serum and aorta lipid concentrations are small, albeit significant, and concentrations are still higher than those of animals on the normal (i.e., low-fat) diet (7, 9, 16). Furthermore, neither of these proposed mechanisms would explain the observed decrease in blood pressure and hypertension with silicon supplementation (12).

We investigated the effect of dietary silicon on a model of diet-induced fatty streak lesions in female apoE knockout mice (C57BL/6J-ApoE/J) fed a diet high in butter fat. We investigated *1*) the development of aortic lesions and blood FA concentrations, *2*) the main structural components of the aorta (collagen and elastin concentrations as well as elastic structure and morphology), and *3*) the biomechanic properties of the aorta (tensile strength and elasticity), to probe potential mechanisms of action of silicon in vascular health. The overall objective of the study was to show whether dietary silicon could protect against atherosclerosis in a model with a strong genetic drive as has been reported for silicon activity in a pure dietary model of atherosclerosis (8).

## Methods

### Materials

The formulated high-fat diets were produced by Dr. Forrest Nielsen (USDA Grand Forks Human Nutrition Research Center, Grand Forks, North Dakota). Monomethylsilanetriol (115-μg silicon/mL) was from LLR-G5 Ltd. (Castlebar, Ireland). Sterile, low-silicon-containing deionized drinking water was from Sigma-Aldrich Co. For sample analysis, ultra-high purity water was 18 mol/LΩ/cm, from a Branstead Nano-Pure water purifier (Thermo Scientific). Ketamine HCL (Ketasol-100) was from Dr. E Graeub AG, and xylazine (Rompun) was from Bayer HealthCare AG. Polypropylene tubes (13 mL and 25 mL) and microvettes (CB 300Z) were from Sarstedt Ltd. Eppendorf tubes (1.5 mL) were from Eppendorf. PBS was from PAA Laboratories GmbH.

### Mice and dietary intervention groups

Female C57BL/6J-ApoE/J mice, referred hereafter as female apoE knockout mice, were obtained from the Institute of Zoology and Genetics (Himberg, Austria). Female mice were chosen because previous studies have shown that the silicon effect is more enhanced in females of the species, implying that there may be a silicon-estrogen interaction (17, 18). The mice were housed in plastic cages with stainless steel covers, at 22°C, with a 12/12-h light/dark cycle and 45% humidity. They were maintained on R/M-H feed (Sniff) and tap water for 6–8 d to acclimatize before being randomly divided into 3 groups of mice (*n* = 9–15 mice/group). The first group, the silicon-deprived (−Si)^9^ group, received a specifically formulated high-fat diet (21% wt anhydrous milk fat and 1.5% wt cholesterol; **Supplemental Table 1**) with low silicon (<3 μg silicon/g) as well as low-silicon drinking water (0.04 μg silicon/mL) ad libitum. The second group was the silicon-replete (+Si) in feed (+Si-feed) group, which received the same high-fat feed but the feed was replete in silicon at 100-μg silicon/g feed, as sodium metasilicate. This is still relatively low in silicon compared to normal murine laboratory maintenance feed (R/M-H), which was found to contain 669 ± 60-μg silicon/g feed. However, sodium metasilicate is readily bioavailable unlike much of the silicon naturally present in laboratory rodent diets (19). Drinking water consisted of the low-silicon water. The third and final group was the silicon-replete in drinking water (+Si-water) group, which received the same high-fat, low-silicon feed as group 1, but their drinking water was replete in silicon at 115 μg silicon/mL in the form of monomethylsilanetriol [CH_3_Si(OH)_3_]. Monomethylsilanetriol was used in the original study that reported the antiatherosclerotic action of silicon (8). It is also safe, metabolized in vivo to orthosilicic acid, Si(OH)_4_ (20, 21), and is a convenient form of soluble silicon for dosing unlike orthosilicic acid because it does not polymerize at the concentration used here.

### Studies 1 and 2

Two studies were conducted. The first study (Study 1) investigated the effect of the dietary silicon on the formation of fatty streak lesions (early atherosclerotic plaques). The −Si group consisted of 9 mice, whereas the +Si groups consisted of 10 mice. Mice were 29 ± 13 d (4–6 wk) old at the start of the intervention. Dietary intervention lasted for 15 wk. One mouse in the −Si group was killed at 11 wk, with an overdose of ketamine hydrochloric acid and xylazine, to check for the development of fatty streak lesions. This was found to be low, so the remaining mice were maintained on their respective diets for an additional 4 wk. One mouse in the −Si group died during the study (week 8). The cause of death was unclear, but it is unlikely to be related to the treatment received. This left only 7 mice in the −Si group at the end of the study.

The second study (Study 2) investigated the effect of the dietary silicon on total serum lipids and on the structural and biomechanic properties of the aorta (tensile strength, elasticity, collagen and elastin content, and elastin structure and morphology). Groups consisted of 15 mice in the −Si group and 10 mice in the +Si groups. Mice were 114 ± 9 d (16–17 wk) old at the start of the intervention and were maintained on their respective diets for up to 19 wk.

In both studies, 5 mice were collected before separation into the 3 dietary silicon groups. The “baseline” samples from these mice will indicate the magnitude of the changes (e.g., in plasma lipid concentrations) caused by the dietary interventions. However, because of the small numbers, these baseline samples were not compared statistically with the treatment groups.

### Study design

Mice were maintained on their respective diets for up to 19 wk as detailed above. Their outward appearance was checked every second day and any differences or abnormalities documented. They were weighed weekly at 0900 on a precision scale (PAC 3000; Sartorius), accurate to 0.001 g, after overnight feed deprivation. A blood sample was collected monthly from the tail vein, again at 0900 after overnight feed deprivation. Overnight feed deprivation consisted of removing their feed and drinking water at 1700 (or earlier) the previous day with only access to deionized water of negligible silicon content throughout the period of feed deprivation. Feed and water intakes were measured as described below. At the end of the dietary intervention period, the mice were killed over a 5-d period to allow time for tissue processing. Equal numbers of mice from the 3 dietary groups were killed on each day. Before killing, mice were deprived of food overnight as described above. At 0900 the following day, they were anesthetized with a mixture of ketamine hydrochloric acid and xylazine, weighed, the peritoneal cavity opened, and a blood sample collected from the abdominal vena cava. Thereafter, mice were immediately killed with an overdose of the ketamine hydrochloric acid and xylazine mixture, and tissues were collected into individual pre-weighed 13-mL polypropylene tubes (kidneys, spleen, liver, lungs, small and large intestines, femurs and tibias, skin, and ears). The aorta and the heart were then harvested. The whole aorta (i.e., the ascending, descending, and abdominal aorta), the aortic arch, and the iliac bifurcation and parts of the common iliac arteries (**Supplemental Figure 1**) were removed and cleaned of connective tissue and fat. The aorta and heart were kept moist with saline throughout the dissection to avoid dehydration and loss of integrity. The heart was then separated from the aorta and placed into a pre-weighed 13-mL polypropylene tube. The aorta was prepared for the analyses as described below. All excised tissues were weighed in pre-weighed polypropylene tubes immediately after collection to determine mass (to the nearest 0.001 g) and snap frozen in liquid nitrogen. Samples were stored at −80°C until analyses. The studies described here were approved by the Commission for Animal Testing of the Austrian Ministry for Science and Research.

### Silicon intake

Feed and water intakes were measured at 9 and 11 wk in Study 1 and at 1 and 15 wk in Study 2. The feed tray and plastic water bottle of each cage were appropriately filled and weighed to the nearest 0.1 g and 0.01 g, respectively, and placed back in the cages. Twenty-four hours later the feed trays and water bottles were reweighed to measure the loss in mass because of intakes. The feed trays and water bottles were refilled and reweighed, and this 24-h measurement was repeated the following day.

### Monthly blood collection

Blood samples were collected monthly from mice in the 3 dietary silicon groups. Mice were deprived of food overnight and were warmed with a heating lamp for up to 5 min and their tails for an additional 2 min before blood collection to ensure good blood flow. A wound was then created on the tail with a scalpel and blood was collected with microvettes. A maximum of 500 μL per mouse was collected.

### Preparation of blood samples

Blood samples were allowed to clot at room temperature for up to 2 h before centrifuging at 9224 × *g* for 10 min at room temperature. The separated serum was collected into Eppendorf tubes and stored at −80°C until analysis.

### Analyses

A brief summary of the analyses is given below with more details in the **Supplemental Materials and Methods**.

#### Fatty lesions (atherosclerotic plaques).

The whole aortas collected from Study 1 mice were quantified for fatty lesions after fixation with paraformaldehyde and staining with Sudan IV. Digital images were collected of the stained aortas, and these were assessed and quantified for the degree of red staining. The mean data of the 7–10 aortas per group are given for each dietary silicon group. Staining and image analysis were carried out in a blinded fashion.

#### Biomechanic properties of the aorta.

The descending aortas collected from Study 2 mice were each segmented into seven 2-mm aortic rings and each underwent biomechanic testing to determine elasticity (or stiffness) and the forces required to rupture the aortic specimen (**Supplemental Figures 2** and **3**). A sensitive tensile testing setup was constructed for these analyses (M Stoiber, H Schima, unpublished results, 2013). Values are reported for each of the 7 individual 2-mm rings. Again, analysis was conducted in a blinded fashion.

#### Elastin and collagen contents.

Total elastin and collagen contents were determined for the aortas collected in Study 2. The descending aortas were air-dried and homogenized, and elastin and collagen were extracted and quantified with use of the Fastin Elastin Assay kit and hydroxyproline assay (22), respectively. Elastin and collagen contents were expressed as gram per gram dry weight of sample. It was assumed aortic collagen contains a mean of 13.5% w/w hydroxyproline (23, 24).

#### Histochemical analysis of the elastic fibers.

The quality of the elastic fibers of the aorta of mice from the 3 dietary silicon groups was examined. The abdominal aorta, aortic arch, and iliac bifurcation (Supplemental Figure 1) from mice in Study 2 were fixed with formalin and embedded in paraffin wax. Four-micrometer thickness sections were stained for the elastic fibers, digitally imaged, and viewed by a pathologist in a blinded fashion.

#### Serum lipids.

Total, HDL, and LDL cholesterol as well as TG concentrations in the terminal serum sample were compared between the 3 dietary silicon groups. Serum samples from (feed-deprived) mice at baseline (before separation into the experimental groups) were similarly analyzed; however, because of the small numbers (*n* = 3), the baseline samples were not compared statistically with the treatment groups. Analysis was conducted with a serum chemistry analyzer (Siemens Healthcare AG) in a blinded fashion, and concentrations are given in mmol/L.

#### Total elemental analysis.

The total silicon concentration in serum from mice in the 3 dietary silicon groups was measured by inductively coupled plasma optical emission spectroscopy, as previously described (25, 26), with use of appropriate sample-based standards. Feed and drinking water samples were also analyzed for total silicon with appropriate standards.

### Statistical analysis

Results are expressed as means ± SDs, unless otherwise stated. All 3 dietary silicon groups were compared with each other. One-factor ANOVA was used to compare means for feed intake, water intake, serum lipid concentrations, deposition of fatty lesions, aortic circumference, aortic collagen concentration, and aortic elastin concentration. Significant difference was taken as *P* < 0.05. The independent samples Kruskal-Wallis test with pairwise comparison was used to assess the means for silicon intake, total serum silicon concentration, and aorta silicon concentration because these data were not normally distributed. Significance was taken as *P* < 0.05. Repeated-measures ANOVA was used to assess body weight over the study period and biomechanic measures (i.e., maximum tear force, elasticity, and circumference) along the 7 aortic positions, with significant group differences taken as *P* < 0.05. Bonferroni post hoc analysis was used to determine the significant differences in maximum tear force. All statistical analysis was conducted in IBM SPSS Statistic 22 for Windows.

## Results

### 

#### Silicon intake.

There was no difference in feed and water intakes between the 3 dietary silicon groups in both studies (**Supplemental Table 2**). In Study 1, silicon intakes in the −Si group were 98.1% lower than the +Si-feed group (*P* < 0.001) and 94.7% lower than the +Si-water group, although this difference was not significant (*P* = 0.07). Overall, feed intakes were lower for the mice in Study 2 than Study 1 but, still, the −Si group had 98.5% lower silicon intake than the +Si-feed group (*P* < 0.001) and 97.5% lower intake than the +Si-water group (*P* = 0.007). Silicon intakes did not differ between the +Si groups in both studies.

#### Body and tissue weights.

**FIGURE 1:**
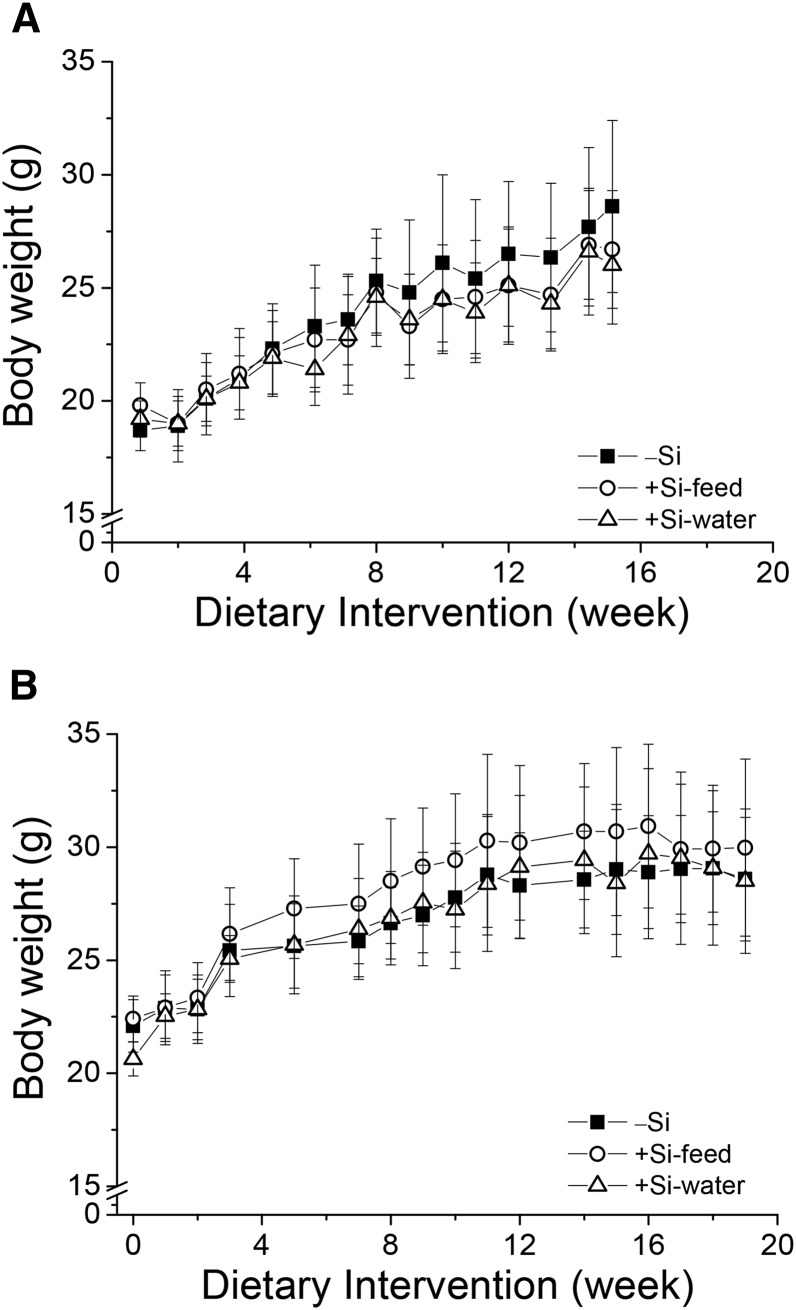
Body weights of female apoE knockout mice in Study 1 (A) and Study 2 (B) fed a diet high in butter fat and depleted in silicon, compared with mice fed the 2 silicon-replete, high-fat diets: silicon was replete in the feed or the drinking water. Values are means ± SDs; *n* = 7–10 for Study 1, and *n* = 10–15 for Study 2. Mice were deprived of food overnight before measurement of body weight. There were no differences in body weight between the 3 dietary silicon groups in both studies. −Si, silicon-deprived; +Si-feed, silicon-replete in feed; +Si-water, silicon-replete in drinking water.

Silicon deprivation had no effect on body mass (**Figure 1**) or the wet mass of the tissues and organs collected at necropsy (**Supplemental Figure 4**); both were comparable between the 3 dietary silicon groups. Equally, there was no difference in outward appearance of the mice over the study period, although some mice in all groups suffered from some hair loss caused by overgrooming, which is common in this strain of mouse.

#### Serum and aorta silicon concentrations.

**FIGURE 2:**
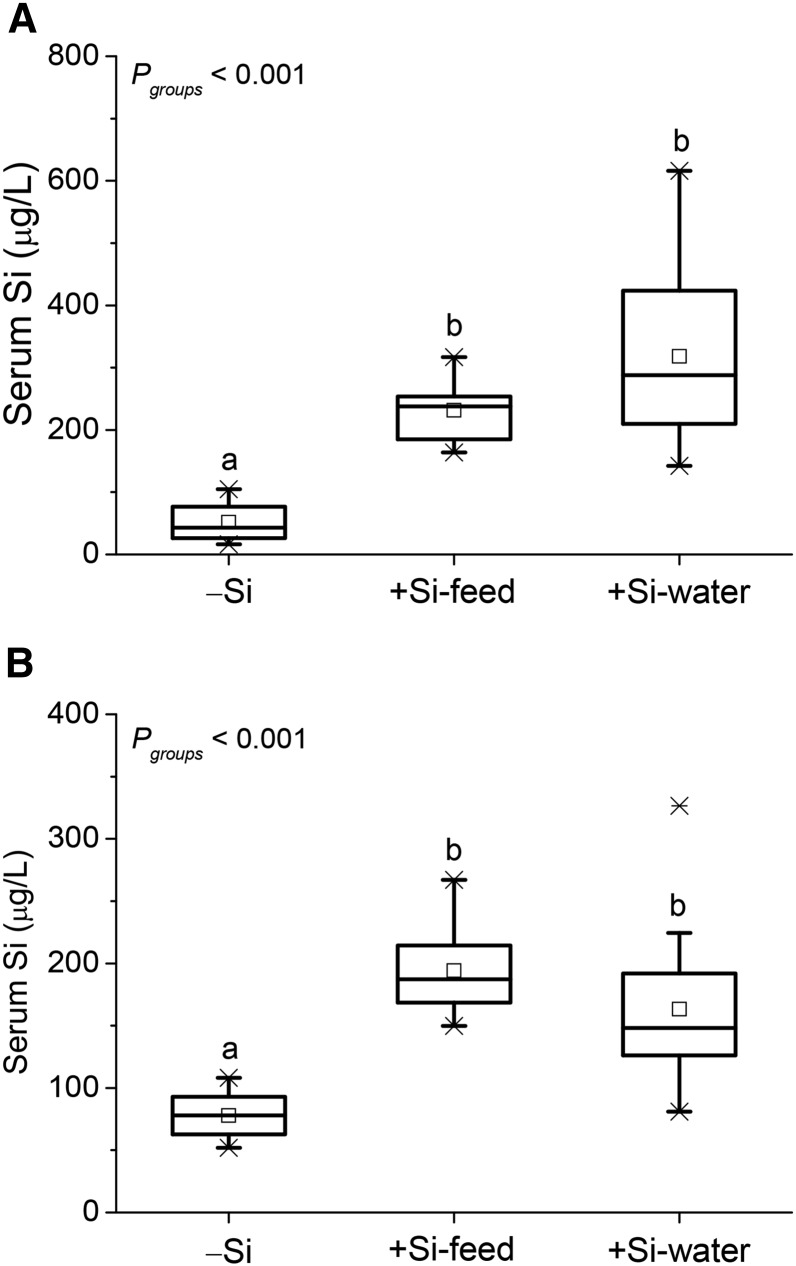
Total silicon concentration in the serum of female apoE knockout mice in Study 1 (A) and Study 2 (B) fed a diet high in butter fat and depleted in silicon, compared with mice fed the 2 silicon-replete, high-fat diets: silicon was replete in the feed or the drinking water. Values (*n* = 7–10/group for Study 1, and *n* = 10–15/group for Study 2) are shown as box-plots where the horizontal lines indicate the 5th, 25th, 50th (i.e., median), 75th, and 95th percentiles, the open squares show the means, and the crosses show the minimum and maximum values. Mice were deprived of food overnight before collection of serum. Within each graph, labeled groups without a common letter differ (*P* < 0.05; independent samples Kruskal-Wallis test with pairwise comparison). Si, silicon; −Si, silicon-deprived; +Si-feed, silicon-replete in feed; +Si-water, silicon-replete in drinking water.

In Study 1, mean serum silicon concentration in the −Si group was 78% lower than the +Si-feed group (*P* = 0.003) and 84% lower than the +Si-water group (*P* < 0.001) (**Figure 2**A). Similarly in Study 2, mean serum silicon concentration in the −Si group was 60% lower than the +Si-feed group (*P* < 0.001) and 52% lower than the +Si-water group (*P* = 0.006) (Figure 2B). There was no difference in serum silicon concentration between the +Si groups.

**FIGURE 3:**
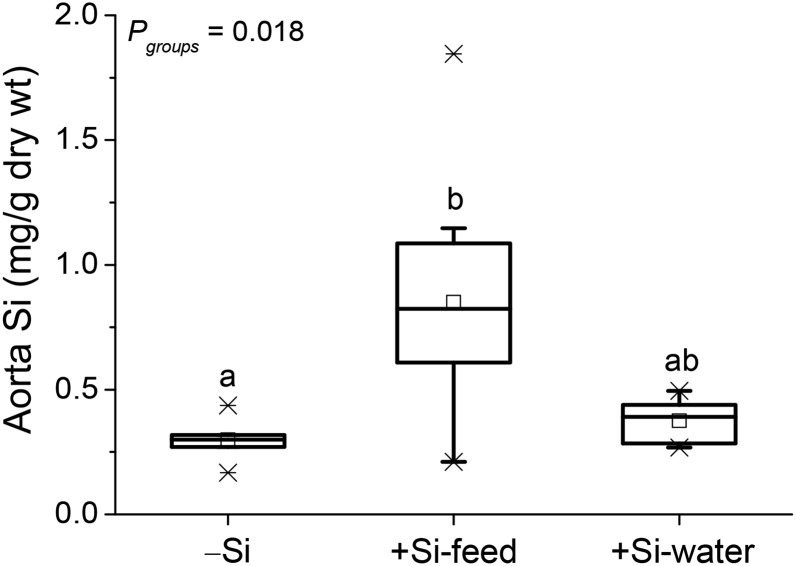
Total silicon concentration of the aorta of female apoE knockout mice in Study 1 fed a diet high in butter fat and depleted in silicon, compared with mice fed the 2 silicon-replete, high-fat diets: silicon was replete in the feed or the drinking water. Values (*n* = 6–10/group) are shown as box-plots where the horizontal lines indicate the 5th, 25th, 50th (i.e., median), 75th, and 95th percentiles, the open squares show the means, and the crosses show the minimum and maximum values. Mice were deprived of food overnight before collection of aorta. Within the graph, labeled groups without a common letter differ (*P* < 0.05; independent samples Kruskal-Wallis test with pairwise comparison). Si, silicon; −Si, silicon-deprived; +Si-feed, silicon-replete in feed; +Si-water, silicon-replete in drinking water.

Silicon concentration of the whole aorta collected in Study 1 was 65% lower in the −Si group than the +Si-feed group (*P* = 0.025), but was not different compared to the +Si-water group (**Figure 3**). Again, there was no difference in (aortic) silicon concentration between the +Si groups.

#### Fatty lesions (atherosclerotic plaques).

**FIGURE 4:**
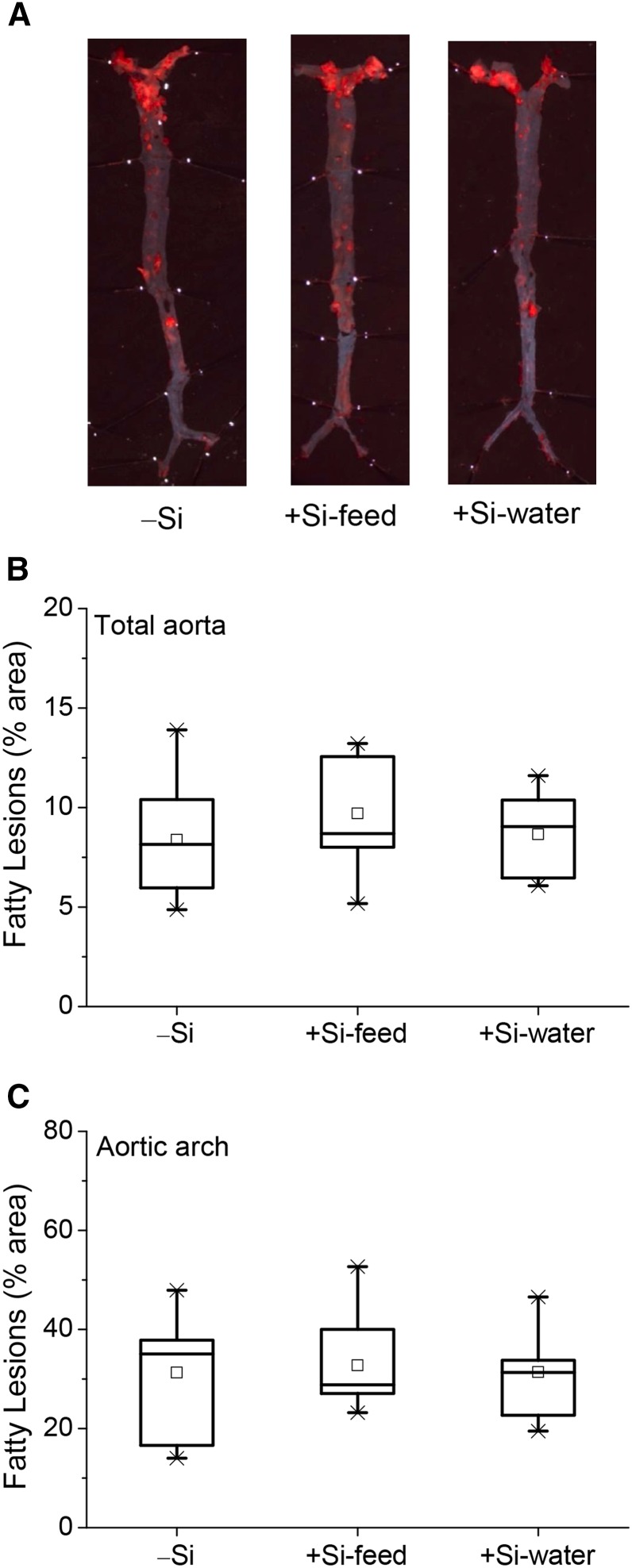
Quantification of fatty lesions in the whole aorta of female apoE knockout mice in Study 1 fed a diet high in butter fat and depleted in silicon, compared with mice fed the 2 silicon-replete, high-fat diets: silicon was replete in the feed or the drinking water. (A) Digital images showing staining of fatty lesions on the luminal surface; one example from each dietary silicon group. Quantification of the whole aortic area occupied by fatty lesions (B) or just at the aortic arch (C), as a percentage of the total area of the aorta quantified. Values (*n* = 7–10/group) are shown as box-plots where the horizontal lines indicate the 5th, 25th, 50th (i.e., median), 75th, and 95th percentiles, the open squares show the means, and the crosses show the minimum and maximum values. There was no difference between the 3 dietary silicon groups. −Si, silicon-deprived; +Si-feed, silicon-replete in feed; +Si-water, silicon-replete in drinking water.

The whole aortas from the mice in Study 1 were stained for fatty lesions (early development of atheromatous plaques) on the luminal surface (**Figure 4**A). However, there was no difference in percentage coverage of the whole aorta (Figure 4B) or the aortic arch (Figure 4C) by fatty lesions between the 3 dietary silicon groups.

#### Serum lipids.

Hemolysis in the serum samples (caused by an accident during processing of the blood) of the −Si group in Study 1 interfered with the lipid analysis of these samples, so comparison with the +Si groups could not be made. However, comparison of serum lipid concentrations (i.e., TGs, total cholesterol, HDL cholesterol, and LDL cholesterol) of the terminal blood samples collected in Study 2 could be made and showed no significant difference between the 3 dietary silicon groups (**Table 1**).

**TABLE 1:** Terminal serum lipid concentrations of female apoE knockout mice fed a diet high in butter fat and depleted in silicon for 19 wk, compared with mice fed the 2 silicon-replete, high-fat diets (Study 2)^1^

		Experimental groups			
	Baseline^2^ (n = 3)	−Si (*n* = 15)^3^	+Si-feed (*n* = 9)^4^	+Si-water (*n* = 10)	*P*^5^
TGs, mmol/L	0.65 ± 0.06	0.85 ± 0.17	0.95 ± 0.15	0.99 ± 0.25	0.17
Total cholesterol, mmol/L	4.87 ± 0.44	8.33 ± 1.33	7.09 ± 0.96	7.76 ± 1.76	0.12
HDL cholesterol, mmol/L	3.00 ± 0.21	4.32 ± 0.56	3.88 ± 0.92	3.98 ± 1.21	0.66
LDL cholesterol, mmol/L	1.58 ± 0.56	2.67 ± 0.63	2.69 ± 0.55	2.86 ± 0.88	0.85

1 Values are means ± SDs; *n* = 3–15 mice/group. Mice were deprived of food overnight before collection of serum. −Si, silicon-deprived (<3-μg silicon/g); +Si-feed, silicon-replete in feed (100-μg silicon/g); +Si-water, silicon-replete in drinking water (115-μg silicon/mL).

2 Female apoE knockout mice before separation into experimental groups and administration of high-fat diet. Baseline samples were not statistically compared to treatment groups.

3 Except HDL cholesterol and LDL cholesterol where *n* = 7.

4 Except HDL cholesterol and LDL cholesterol where *n* = 8.

5 One-factor ANOVA comparison of the experimental groups.

#### Biomechanic tests.

**FIGURE 5:**
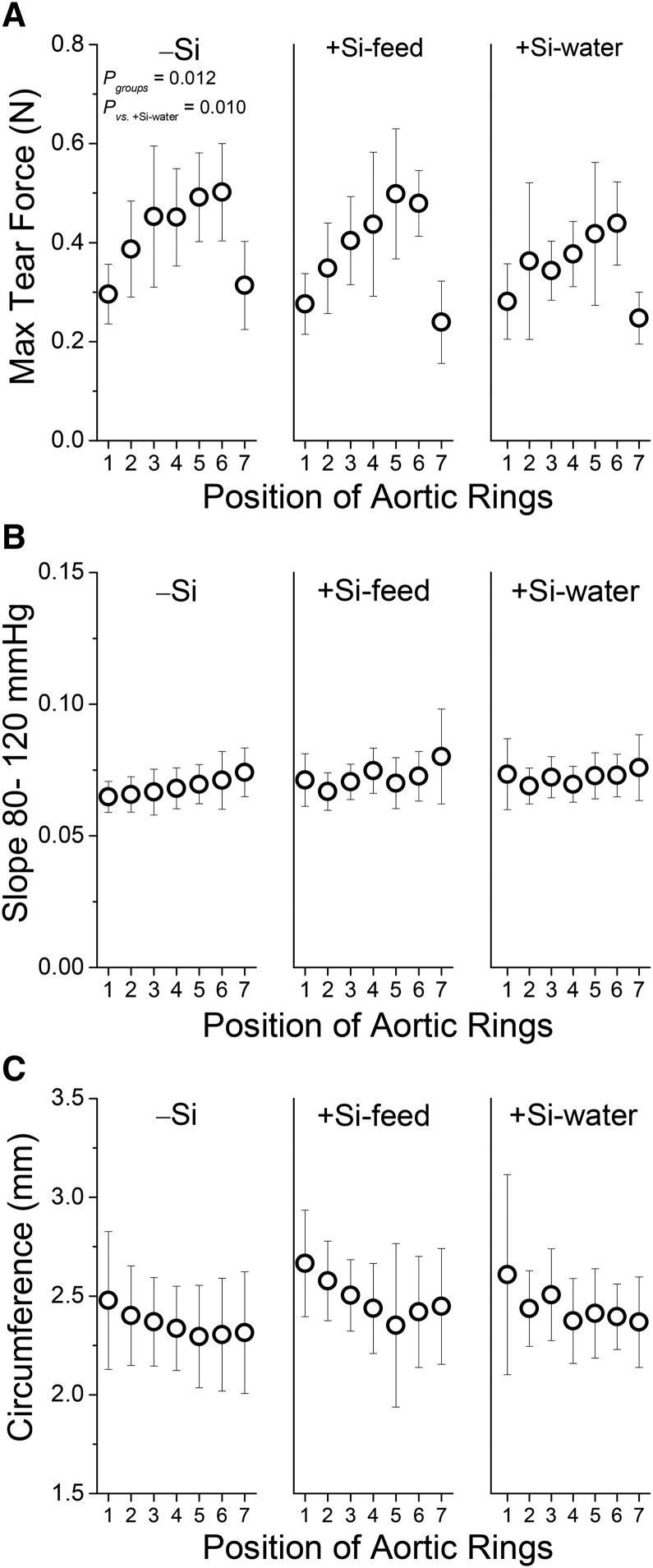
Tensile strength (A), stiffness (B), and initial ring circumference (C) of the aorta of female apoE knockout mice in Study 2 fed a diet high in butter fat and depleted in silicon, compared with mice fed the 2 silicon-replete, high-fat diets: silicon was replete in the feed or the drinking water. Values are shown for each of the 7 aortic rings/positions from each mouse. Values are means ± SDs; *n* = 10–15 mice/position/group. Stiffness (B) and initial ring circumference (C) profiles were similar for the 3 dietary silicon groups. −Si, silicon-deprived; +Si-feed, silicon-replete in feed; +Si-water, silicon-replete in drinking water.

From the tensile measurements in Study 2, the forces required to rupture the aortic wall were determined for the different dietary silicon groups. Maximum tear force increased when moving down the descending aorta, peaking at position 5 or 6 with a reduction at position 7 (**Figure 5**A). The pattern was comparable between the −Si and +Si-feed groups, but a significant difference was found between −Si and +Si-water groups (*P* = 0.010), suggesting less force was required to rupture the aortic wall in the +Si-water group. There was no difference in maximum tear force between +Si groups.

A higher slope was found moving down the descending aorta from position 1 to position 7, i.e., away from the aortic arch and heart (Figure 5B), suggesting that the aorta is less flexible as it moves away from the heart. This pattern did not differ significantly between the 3 dietary silicon groups.

Initial ring circumference at the 7 positions along the aorta was found to decrease from position 1 to position 5 and level off thereafter (Figure 5C). A comparable pattern was seen for the 3 dietary silicon groups. Averaging the circumference at the 7 positions also did not show any significant difference between the 3 dietary silicon groups, even at higher aortic pressures (**Supplemental Table 3**).

#### Elastic structure and morphology.

Histochemical staining and pathologic analysis of the aortic arch, abdominal aorta, and iliac bifurcation from the aortas collected in Study 2 showed no difference in the structure, size, or number of the elastic fibers between the 3 dietary silicon groups (**Supplemental Figure 5**).

#### Collagen and elastin contents.

**FIGURE 6:**
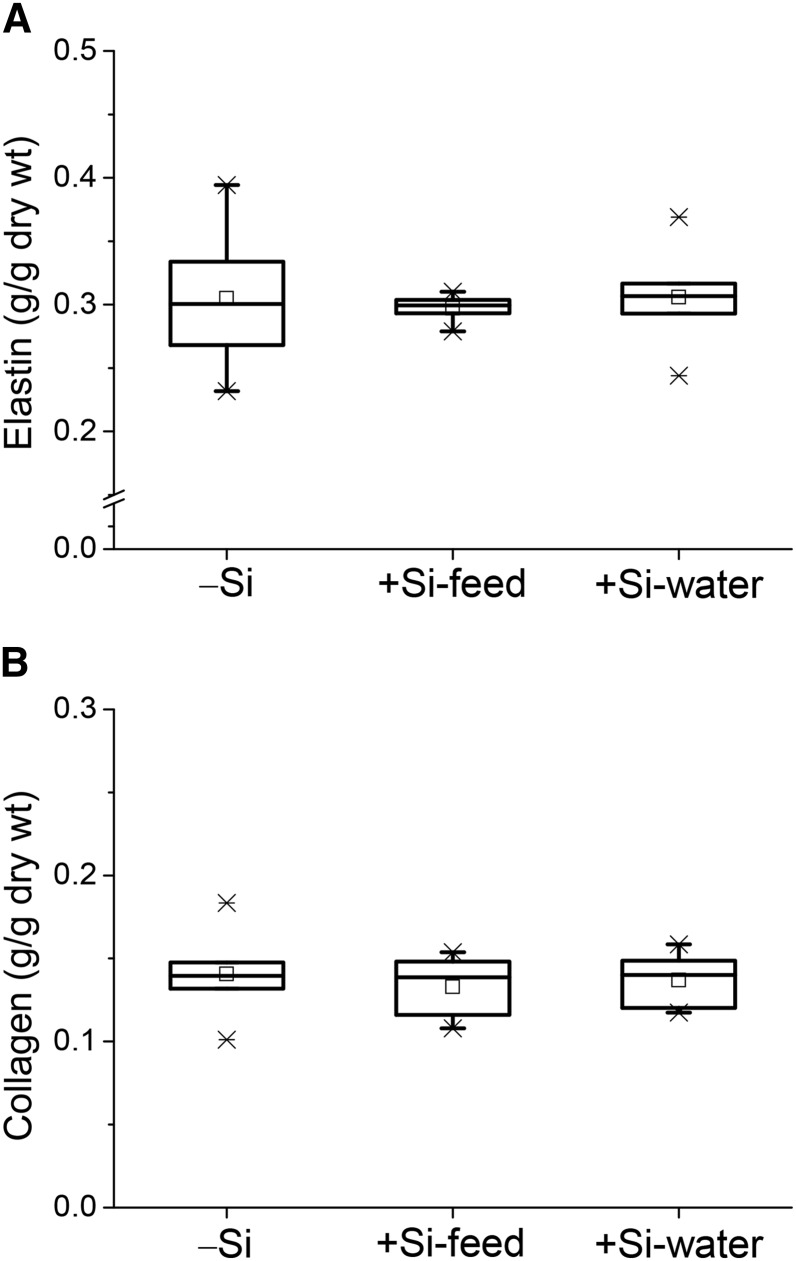
Total elastin (A) and collagen (B) contents of the aorta of female apoE knockout mice in Study 2 fed a diet high in butter fat and depleted in silicon, compared with mice fed the 2 silicon-replete, high-fat diets: silicon was replete in the feed or the drinking water. Values (*n* = 5–6/group) are shown as box-plots where the horizontal lines indicate the 5th, 25th, 50th (i.e., median), 75th, and 95th percentiles, the open squares show the means, and the crosses show the minimum and maximum values. There were no differences in elastin and collagen contents between the 3 dietary silicon groups. −Si, silicon-deprived; +Si-feed, silicon-replete in feed; +Si-water, silicon-replete in drinking water.

Analysis of the descending aortas collected in Study 2 for total elastin and collagen contents found no difference in mean aortic contents between the 3 dietary silicon groups (**Figure 6**).

## Discussion

This study aimed to investigate the mechanism of action of dietary silicon on vascular (aortic) health using a mouse model of atherosclerosis. Overall, we found that dietary silicon deprivation had no effect on the development of fatty lesions or on serum lipid concentrations. Dietary silicon deprivation also had no effect on the elastin and collagen contents of the aorta wall, morphology of the elastin fibers, or the biomechanic properties of the aorta.

The lack of effect of dietary silicon on the development of fatty lesions is contrary to the findings of others including Loeper et al. (8, 9), who reported that dietary silicon supplementation significantly reduced, and almost negated, the formation of atheromatous plaques in a cholesterol-fed rabbit model. This disparity in findings may be explained by the severity of the model used herein. The apoE knockout mouse model used here is a well-established genetic model of atherosclerosis (27, 28), but it is a more severe mouse model than others that exist (29, 30). Notably, the complete lack of lipoprotein clearance in the apoE mice affects other anti-inflammatory and immunomodulatory properties, and these mice also develop diabetes and Alzheimer’s dementia (27, 29, 30). The apoE mice also appear to show a lack of sensitivity toward statins, the standard therapy for patients with atherosclerosis (27, 28, 31–35). The other mouse models (lipoprotein lipase knockout and LDL receptor knockout) are less severe and more specifically target the cardiovascular system (29, 30), but again, these also show differences in sensitivities toward statins and thus do not completely mimic the human condition. The cholesterol-fed rabbit model used by Loeper et al. (8, 9) and others is a much less severe model as judged by the more modest increase in total serum cholesterol/lipids that is observed (7–9). However, even with the limitation described above, the apoE knockout mouse model has been used to investigate the effect of nutritional components on atherosclerosis development, and a reduction in plaque area has been reported for a number of nutrients (36–40). A marked reduction in the plasma total cholesterol concentration was additionally reported by Xia et al. (39) and Mori et al. (40). These studies (36–40) were conducted in male apoE knockout mice ≥6 wk of age, and the mechanism of action of 3 of the compounds (36–39) is described as being related to their anti-inflammatory activities. Other nutrients/nutritional factors have also been tested in the apoE knockout mouse model and shown to be antiatherosclerotic, again related to their anti-inflammatory or antioxidant activities (41–46). Although one study reported that dietary silicon exposure may influence the immune/inflammatory response (47), the lack of activity here may imply that dietary silicon has no direct anti-inflammatory or antioxidant properties in the apoE knockout mouse model. However, this study was not designed to specifically investigate the anti-inflammatory properties of dietary silicon. CLA, present in dairy products including butter [CLA constitutes ∼0.44% of total FAs (48)], may have masked any small effect of silicon on inflammation. Milk (butter) fat was used here because it accelerates atherosclerosis development, is more potent than other sources of fats (49), and has been used previously in the apoE knockout mouse model [e.g., Buus et al. (37)]. Female (apoE) mice were used here because our previous studies suggest females of the species are more sensitive to dietary silicon exposure (17, 18).

One of the proposed mechanisms of action of dietary silicon is that it may decrease the concentrations of FAs in serum and aorta that are negatively linked to atherosclerotic plaque formation (7–9, 16). Loeper et al. (9) reported a 13% drop in total serum lipids, a 15% drop in total serum cholesterol, and a 20% drop in aorta lipid concentration with silicon supplementation (monomethylsilanetriol) in rabbits. Loeper et al. (9) also studied FFAs and total FAs and esters in serum and the aorta and concluded that silicon reduced MUFAs and PUFAs, in particular arachidonic acid, which is involved in lipid peroxidation. Trincă et al. (7) similarly reported a drop in serum cholesterol (by 44%), total lipids (44%), HDL cholesterol (18%), FFAs (31%), TGs (29%), and phospholipids (26%) with silicon supplementation (sodium silicate) in rabbits. Najda et al. (16), however, reported no change in total serum cholesterol, an increase in serum HDL cholesterol (20%) and serum HDL phospholipids (30%), and a drop in serum LDL cholesterol (23%), serum TGs (30%), and aorta cholesterol concentration (20%) with silicon supplementation (sodium metasilicate) in male Wistar rats. These findings suggest that the antiatherosclerotic effects of silicon may be partly dependent on a reduction in serum lipids (16). In our study, no significant difference in serum lipid concentrations was found between the dietary silicon groups (Table 1). Thus, overall we conclude that, in the apoE mouse model, silicon deprivation had no adverse effect on total serum cholesterol and thus no effect on aortic lesions. It should be pointed out that the 2 apoE mouse studies that reported both a marked reduction in plasma total cholesterol and a reduction in aortic fatty lesions used a normal diet containing 5–7% fat and <0.07% cholesterol (39, 40). The diet used here had a higher fat content (21% wt milk fat) and a higher concentration of cholesterol (1.5% wt).

It has also been suggested that silicon is an important structural component of the aortic wall that protects the vessel against lipid permeability and the development of fatty lesions. Collagen and elastin are 2 of the major components of the aortic wall that determine its biomechanic properties and functional integrity, and in atherosclerosis these are broken down (38, 50). Silicon may bind to these components to increase their stability or it may increase the synthesis and contents of these components in the aortic wall (10). Indeed, Loeper et al. (8) reported that the elastic fibers seemed “undamaged and often thickened” in the silicon-supplemented group in the cholesterol-fed rabbit model. Additionally, statins have been suggested to increase the collagen content of the aorta to stabilize the plaques and reduce their rupture (30). Here, we investigated whether silicon deprivation could alter the composition of collagen and elastin in the aortic wall and thereby decrease the stability of the aorta. Silicon deprivation had no effect on total elastin and collagen contents or morphology of the elastic fibers. Macroscopically, the elastic fibers in all the groups looked “normal,” suggesting that the aortas were not markedly damaged in our studies. Santelices et al. (51) showed that damage to the aorta in apoE mice is slow but progressive until between 25 and 34 wk of age when a marked increase in damage to the aortic wall is observed. The apparent lack of difference in composition of the aortic wall between the 3 different dietary silicon groups is further confirmed by the similarities in the biomechanic properties of the aorta.

It is possible that, although serum silicon concentrations were lower in the −Si group, than in the +Si groups, the mice may not have been deficient in silicon. Indeed, the lack of significant difference in aortic silicon concentrations between the −Si and +Si-water groups would imply this to be the case, and this could explain the lack of difference in structural and biomechanic properties of the aortas between the dietary silicon groups. We previously reported that female Sprague Dawley rats on a −Si diet actively maintained their tissue silicon concentrations by reducing/inhibiting urinary silicon output (3), and so the same could have occurred in the mice in this study.

In conclusion, we found that dietary silicon deprivation had no effect on the development of atheromatous lesions in the apoE knockout mouse model. This is contrary to the reported findings in the cholesterol-fed rabbit model and the positive effects of dietary silicon supplementation on vascular health reported by others. Further work, in a dietary-based rather than “genetically programmed” model of atherosclerosis, is now needed to corroborate these findings.

## Supplementary Material

Online Supporting Material

## Footnotes

9 Abbreviations used: −Si, silicon-deprived; +Si, silicon-replete; +Si-feed, silicon-replete in feed; +Si-water, silicon-replete in drinking water.
