# Supplementary material for: Dietary Silicon Deficiency Does Not Exacerbate Diet-Induced Fatty Lesions in Female ApoE Knockout Mice
Source: J Nutr. 2015 May 13;145(7):1498–506. doi: 10.3945/jn.114.206193 (PMC4478943; doi:10.3945/jn.114.206193)
Supplement: Online Supporting Material [file supp_145_7_1498__index.html]

Dietary Silicon Deficiency Does Not Exacerbate Diet-Induced Fatty Lesions in Female ApoE Knockout Mice — Dietary Silicon Deficiency Does Not Exacerbate Diet-Induced Fatty Lesions in Female ApoE Knockout Mice — Online Supporting Material 

# Dietary Silicon Deficiency Does Not Exacerbate Diet-Induced Fatty Lesions in Female ApoE Knockout Mice

## Online Supporting Material

- Online Supporting Material - Text, Tables 1-3, and Figures 1-5
