# Supplementary material for: Dietary Silicon Deficiency Does Not Exacerbate Diet-Induced Fatty Lesions in Female ApoE Knockout Mice
Source: J Nutr. 2015 May 13;145(7):1498–506. doi: 10.3945/jn.114.206193 (PMC4478943; doi:10.3945/jn.114.206193)
Supplement: Online Supporting Material [file jn.114.206193_nutrition206193SupplementaryData1.docx]

**Supplemental Materials and Methods**

More thorough methodological details on the analyses performed in the paper are given below.

**Materials**

Ultra high purity (UHP) water was 18 MΩ/cm, from a Branstead Nano-Pure water purifier (Thermo Scientific; Ohio, USA). Phosphate buffered saline (PBS) was from PAA Laboratories GmbH (Austria). Butylated hydroxytoluene (BHT) and Sudan IV were from Sigma-Aldrich Co. (Germany), whilst sucrose was Sigma-Aldrich Co. (USA). Paraformaldehyde (PFA), acetone, ethanol (100%) and ethylenediaminetetraacetic acid (EDTA) were from Merck KGaA (Germany). Hypodermic needles (0.4 and 0.7 mm) were BD Mircolane TM (BD Systems Ltd, UK). Acupuncture needles (AkuSensitib 0.2 x 15 mm) were from Karl Blum GmbH (Germany). Xylene was from Thermo Fisher Scientific, UK. Hematoxylin solution, ferric chloride solution, Weigerts Iodine solution, Van Gieson solution, elastin from bovine neck ligament, *trans*-4-hydroxy-L-proline, bovine skin gelatine powder, isopropanol (99%, molecular biology grade), chloramine-T trihydrate (99%, ACS grade) and hydrochloric acid (37%, ACS reagent) were all from Sigma-Aldrich Co. (Dorset, UK). Oxalic acid, Erlich’s Reagent (4-dimethylamino benzaldehyde (4-DABA), > 99% purity) and high purity nitric acid (69% (w/v) p.a. plus) were from Fluka Ltd (Gillingham, UK). Silicon ICP stock standard solution (1,000 mg/L Si) was from VWR Ltd (Poole, UK). Fastin™ Elastin Assay kit was from Biocolor Ltd (Antrim, UK). 96-well microwell plates were Costar (Corning Lifesciences, USA).

**Analyses**

*Fatty lesions (atherosclerotic plaques)*

The whole aortas collected in Study 1 were quantified for fatty lesions (atheromatous plaques). The aortas were carefully cleaned of all adherent fat on the external vessel walls and rinsed thoroughly with PBS-based wash solution (pH 7.4) containing 20 µM BHT and 2 µM EDTA, before being carefully opened longitudinally and pinned with acupuncture needles to expose the luminal surface. Samples were fixed overnight at 4°C with a 5% sucrose solution (pH 7.4) containing 4% PFA and 20 µM EDTA. Excess fixing solution was then removed and the aortas incubated with 0.5% Sudan IV in 35% ethanol and 50% acetone for 15 minutes at room temperature to stain any fat deposits on the luminal surface red. The aortas were then washed with 75% ethanol to remove any non-specific staining and images collected with a surgical microscope equipped with a camera and documentation system (Leica Microsystems, Germany). The collected digital images were subjected to image analysis to quantify the degree of staining.

The digital images were analyzed in Adobe Photoshop CS3 (Adobe Systems Inc., Germany). Using the hand drawing tool, a trace was carefully made around the aorta to outline (crop) the whole aorta for analysis from the digital image. The total number of pixels in the outline was measured, as a measure of the total tissue area. Various other parameters were measured, including area, circumference, height, broadness and density of the pixels. In the second step of the image analysis, the number of red pixels in the outline was measured, as a measure of the area covered by fat deposits (fatty streaks). As before, area, circumference, height, broadness and density of red pixels were also measured. To determine the proportion of atherosclerotic plaques in the outline (aorta), the number of red pixels was divided by the total number of pixels in the outline and multiplied by 100. This was calculated for each mouse in the three dietary intervention groups and the data (n = 7 or 10, per group) averaged for each group. Staining and image analysis were carried out in a blinded fashion.

*Mechanical properties of the mice aortas*

For biomechanical investigations the descending aortas were segmented into seven rings of 2 mm height (**Supplemental Figure 1**). These aortic rings are referred as positions 1 to 7, with position 1 being closest to the aortic arch and position 7 being furthest away and close to the renal arteries. To maintain the functional integrity of the aorta it was kept moist with PBS-saline during its collection, processing and analysis. A BOSE® Electro Force test bench system with a frictionless linear motor with integrated displacement transducer (Type LM1, Bose Corp. MN, USA) was used. The system was adapted with a 0.5 N load cell (Model 31, Sensotec Inc. OH, USA) and a custom made cantilever system to allow fixation of these very small ring specimens (Stoiber M *et al*. unpublished data). The initial preload was set to 0.003 N, which is approximately equivalent to an intravascular pressure of 15 mmHg. The circumferential load was applied via two pins (see **Supplemental Figure 2**) at a constant speed of 1.14 mm/min until rupture. Strain and the resulting force were recorded with the Bose® system at a variable sample rate which records change in force of 0.001 N and change in displacement of 0.001 mm (Bose proprietary “level-crossing sampling”).

Maximum tear force as well as slopes of the force-strain curves (**Supplemental Figure 3)** at arterial pressures between 80 and 120 mm mercury were calculated to determine the elasticity of each aortic ring. The initial ring circumference was estimated at initial preload by taking into account the pin diameter and the pin distance.

*Elastin and collagen contents*

The descending aortas from Study 2 mice were air dried, weighed, pooled (each pooled sample consisted of the aortas from two mice of the same group) and homogenized in 1 mL of 0.25 M oxalic acid with a 9 W sonicator probe (Misonix Microson XL2000 Ultrasonic Cell) for 1 min at room temperature. The suspension was then incubated at 98°C on a heating plate (Dri-Block DB-1; Techne) for 1 h and the supernatant (oxalic acid extract, OAE), containing solubilized α-elastin and collagen was collected by centrifugation (Heraeus Fresco 17; Thermo Scientific) at 11,000 x g for 20 min. The residual tissue in the tube was further incubated with 0.75 mL 0.25 M oxalic acid at 98°C for 1h. Up to four OAE were required to completely solubilize the insoluble elastin present in the aorta samples. The OAEs collected from the various extractions were pooled together (pOAE) and aliquots were analyzed for total elastin and collagen contents.

Total elastin content, i.e. soluble tropoelastin and mature insoluble elastin, were determined with the Fastin™ Elastin Assay kit. This assay only detects elastin and is unaffected by the presence of collagen in the pOAE. The assay was carried out in triplicate with 200 μL aliquots of the pOAE from each sample; n = 6 samples in the Si-deprived group and n = 5 samples each in the Si-replete groups. The final dye solution was placed in a 96-well microplate and the optical density was measured at 540 nm using an optical microwell plate reader (Labsystems Multiskan RC, Artisan Scientific, Champaign, IL, USA). Elastin content was calculated from a standard curve produced with α-elastin (5 to 70 μg) from bovine neck ligament. Elastin content in the samples was expressed as g elastin/g dry tissue weight.

Total hydroxyproline (OH-Pro) content of the pOAE was used to estimate the amount of collagen in the aorta samples (23). Aliquots (200 μL) of the pOAE, containing collagen and other matrix proteins, was freeze dried (LTE Mini Lyotrap, LTE Scientific Ltd, Oldham, UK) for 24 h in 0.3 mL crimp-top borosilicate glass vials (Chromacol, UK) and hydrolyzed by vapor phase HCl in a CEM Discover Protein Hydrolysis system (CEM; Matthews, NC, USA). Samples were hydrolyzed at 150°C for 15 min under anaerobic conditions (15 psi nitrogen). Vapor phase HCl was generated from 10 mL 6 M HCl added to the reaction vessel. The hydrolyzed samples were reconstituted in 50 μL 50% isopropanol and 10 μL aliquots transferred to a 96-well microwell plate and mixed with buffered chloramine-T reagent for 5 min at room temperature. 0.1 mL of Erlich’s Reagent was then added and the chromophore allowed to develop for 30 min at 60°C before measurement of absorbance at 540 nm with an optical plate reader (Labsystems Multiskan RC). *Trans*-4-hydroxy-L-proline standards (0 to 0.25 mg/mL) were used to determine hydroxyproline content in the samples and bovine skin gelatin was used as a quality control. Collagen content was expressed as g of collagen per g dry weight of sample, assuming that collagen contains an average of 13.5% w/w hydroxyproline (24,25).

*Histochemical analysis of the elastic fibers*

Morphological features of the elastic fibers of the internal elastic lamina, of the aorta samples collected in Study 2, was investigated as follows. The bottom half of the abdominal aorta and aortic arch (Supplemental Figure 1) were fixed with 4% formalin at 22°C for 1-3 days (depending on the day of their excision). The formalin-fixed aortas were then embedded in paraffin wax and sectioned at 4 µm thickness using a rotary microtome (Leica Microsystems, Germany). Cross-sections of the aortic arch, abdominal aorta and the iliac bifurcation (i.e. bottom end of the abdominal aorta; Supplemental Figure 1) were obtained. To ensure that the three segments of the aorta from each mouse were stained under the same conditions, all three cross sections from the same mouse was placed on the same glass slide. Sections were de-paraffinized with two changes of xylene for ten minutes each at room temperature and then rehydrated in ethanol solutions of decreasing concentration and, finally, distilled water. De-paraffinized sections were carefully washed with distilled water and, via regressive staining, over-stained with Elastin working solution (containing 20 mL hematoxylin solution, 3 ml ferric chloride solution, 8 ml Weigert’s solution and 5 mL distilled water) for ten minutes at room temperature. Stained sections were decolorized, according to the manufacturer’s instructions (Sigma Aldrich Germany), with dilute ferric chloride solution (3 mL diluted with 37 mL distilled water) and washed with 95% ethanol to eliminate iodine. Decolored sections were stained with Van Gieson solution for 1-3 minutes and washed with 95% ethanol. Washed sections were left in 100% ethanol for 5 minutes before being placed in xylene for another five minutes. Sections were fixed with permanent mounting media and covered with a cover-slip. Digital images were obtained for each segment (e.g. aortic arch, abdominal aorta and iliac bifurcation) from a minimum of two mice per group with an Olympus microscope equipped with a digital camera. For the aortic arch a magnification of ×4 was used, for the middle segment and the iliac bifurcation a magnification of ×20 was used. Structure and number of elastic lamellae were compared between the dietary Si groups. The digital images were also viewed independently by a pathologist in a blinded fashion.

*Serum lipids*

Terminal serum samples collected at sacrifice from overnight feed deprived mice from the two studies were analyzed for total serum lipids. Total and high density lipoprotein (HDL) -cholesterol and triglycerides concentrations were measured on a Siemens Dimension Xpand Chemistry analyzer (Siemens Healthcare AG; Germany). Low density lipoprotein (LDL) -cholesterol was calculated from these concentrations using the Friedewald equation, which assumes the concentration of very low density lipoprotein (VLDL) -cholesterol is equal to triglycerides concentration (mol/L) / 2.2. Due to the high HDL-cholesterol concentrations in some samples, especially from Study 1, the serum samples were diluted 1:2 to allow the determination of HDL-cholesterol concentration in these samples. Analysis was conducted in a blinded fashion and concentrations are given in mmol/L.

*Total elemental analysis*

Total analysis for Si was carried out by inductively coupled plasma optical emission spectroscopy (ICP-OES; Jobin Yvon Ultama-2C, Instrument SA, Longjumeau, France) at 251.611 nm. The ICP-OES was equipped with a concentric nebulizer, cyclonic spray chamber and a low-flow sample uptake tubing (sample flow rate was 0.2 mL/min). Peak profiles were used as previously described (26,27), with a window size of 0.08 nm (0.04 nm either side of the peak) with 21 increments per profile and an integration time of 0.5 second per increment.

To obtain the required volume of sample to undertake a serum Si analysis for each mouse, the monthly collected tail vein serum samples (collected following overnight feed deprivation) were pooled for each mouse. Thus for each mouse in study 1, their tail vein serum samples, collected on days 6, 14, 35 and 105, were pooled. Similarly, for Study 2, the serum samples collected on days 108 and 133 were pooled for each mouse. The serum samples from study 1 and 2 were diluted, at 1+4 and 1+5 respectively, with 0.2% nitric acid (prepared by diluting 69% nitric acid in UHP water) in 13 ml PP tubes. These diluted samples were analyzed with standards prepared by spiking aliquots of similarly diluted rat sera with Si (0-200 ppb). Silicon standards (0-200 ppb) were also prepared in 0.2% nitric acid to determine the Si content of the acid diluent. Diluted sera and acid diluent (0.2% HNO_3_) were analyzed with their appropriate set of standards in a single batch for each study.

Drinking water samples were analyzed for total Si with standards (0-10 ppm) prepared in UHP water. The formulated high-fat feeds and maintenance feed were also analyzed for Si content. Samples of the feeds (0.05-0.3 g) were digested in nitric acid (17-35%) in a Milestone Ethos Plus microwave digestion system at 180°C (10 min RAMP to 180°C and 15 min at 180°C). Sample blanks were similarly prepared. Digested samples and blanks were analyzed with Si standards (0-10 ppm) prepared in 17% nitric acid.

**SUPPLEMENTAL TABLE 1.** Composition of the formulated high fat, low-Si feed

| **Ingredients** | **g/kg feed** |
| --- | --- |
| Casein (water-washed) | 195 |
| DL-Methionine | 3 |
| Sucrose | 343 |
| Corn Starch (acid-washed) | 150 |
| Anhydrous Milk fat | 210 |
| 2-ambo-α-tocopherol | 0.2 |
| Choline Chloride | 0.75 |
| Cholesterol | 1.5 |
| Avicel Fiber | 50 |
| KH_2_PO_4_ | 10 |
| CaCO_3_ | 12.5 |
| 2-(1,1-dimethylethyl)-1,4-benzenediol (TBHQ) | 0.014 |
| Vitamin Mix | 4.55 |
| Mineral Mix | 20 |
| **Mineral Mix:** |  |
| KCl | 1.5 |
| NaCl | 2.1 |
| Mg(C_2_H_3_O_2_)_2_ •4 H_2_O | 4.4 |
| Mn(C_2_H_3_O_2_)_2_ •4 H_2_O | 0.225 |
| Zn(C_2_H_3_O_2_)_2_ •2 H_2_O | 0.15 |
| FeSO_4_ •7 H_2_O | 0.175 |
| CuSO_4_ •5 H_2_O | 0.02 |
| NaF | 0.002 |
| KI | 0.0002 |
| Na_2_SeO_3_ •5 H_2_O | 0.0005 |
| (NH_4_)_2_MoO_4_ | 0.0005 |
| Cr(C_2_H_3_O_2_)_3_ •2 H_2_O | 0.002 |
| H_3_BO_3_ | 0.006 |
| NH_4_VO_3_ | 0.0003 |
| NiCl_2_ •6 H_2_O | 0.002 |
| Na_2_HAsO_4_ •7 H_2_O | 0.005 |
| Sucrose | 11.4 |
| **Vitamin Mix:** |  |
| Vitamin A Palmitate (500,000IU/g) | 0.016 |
| Chole-calciferol (400,000IU/g) | 0.0038 |
| Menadione | 0.001 |
| Biotin | 0.001 |
| Folic Acid | 0.002 |
| Inositol | 0.05 |
| Niacin | 0.03 |
| D-Calcium Pantothenate | 0.01 |
| Riboflavin | 0.027 |
| Thiamine HCl | 0.01 |
| Pyridoxine HCl | 0.015 |
| Cyanocobalamin | 0.00005 |
| Para-aminobenzoic Acid (PABA) | 0.005 |
| Glucose | 4.38 |

**SUPPLEMENTAL TABLE 2.** Daily feed, water and Si intakes of the female Apo-E knockout mice fed a diet high in butter fat and depleted in Si, compared with mice on the two Si-replete, high-fat diets ^1^

|  | Experimental groups | | | *P* values^2^ |
| --- | --- | --- | --- | --- |
|  | −Si | +Si-feed | +Si-water |  |
| *Study 1* |  |  |  |  |
| Feed (g/(day·g Bwt)) | 0.50 ± 0.18 | 0.57 ± 0.21 | 0.45 ± 0.16 | 0.39 |
| Water (mL/(day·g Bwt)) | 0.16 ± 0.03 | 0.18 ± 0.03 | 0.17 ± 0.06 | 0.57 |
| Si intake (µg/g Bwt) | 1.10 ± 0.36^a^ | 57.4 ± 20.9^b^ | 20.7 ± 7.1^ab^ | < 0.001 |
| *Study 2* |  |  |  |  |
| Feed (g/(day·g Bwt)) | 0.16 ± 0.10 | 0.24 ± 0.10 | 0.21 ± 0.07 | 0.20 |
| Water (mL/(day·g Bwt)) | 0.14 ± 0.04 | 0.16 ± 0.07 | 0.12 ± 0.06 | 0.51 |
| Si intake (µg/g Bwt) | 0.36 ± 0.22^a^ | 24.0 ± 9.7^b^ | 14.6 ± 6.4^b^ | < 0.001 |

^1^Mean ± SD: n = 8 for Study 1 and n = 4 for Study 2

^2^One-way ANOVA, except for Si intake where Independent Samples Kruskal-Wallis Test was used. Labelled means in a row without a common letter differ, *P* < 0.05 (Independent Samples Kruskal-Wallis Test with pairwise comparison).

−Si, Si-deprived group (< 3 µg Si/g); +Si-feed, Si-replete in feed group (100 µg Si/g); +Si-water, Si-replete in drinking water group (115 µg Si/mL).

**SUPPLEMENTAL TABLE 3.** Circumference, at different pressures, of the aortic rings from the female Apo-E knockout mice fed a diet high in butter fat and depleted in Si, compared with mice on the two Si-replete high-fat diets (Study 2)^1^

| Circumference at: | Experimental groups | | |  | *P* values^2^ |
| --- | --- | --- | --- | --- | --- |
|  | −Si  (mm) | +Si-feed  (mm) | +Si-water  (mm) |  |  |
| 0 mm Hg | 2.35 ± 0.25 | 2.49 ± 0.28 | 2.44 ± 0.22 |  | 0.36 |
| 80 mm Hg | 3.39 ± 0.24 | 3.52 ± 0.18 | 3.44 ± 0.23 |  | 0.32 |
| 100 mm Hg | 3.65 ± 0.25 | 3.78 ± 0.17 | 3.70 ± 0.23 |  | 0.35 |
| 120 mm Hg | 3.88 ± 0.25 | 4.00 ± 0.16 | 3.90 ± 0.23 |  | 0.40 |

^1^Means ± SD: n = 10-15 mice/group

^2^Between group differences (One-way ANOVA).

−Si, Si-deprived group (< 3 µg Si/g); +Si-feed, Si-replete in feed group (100 µg Si/g); +Si-water, Si-replete in drinking water group (115 µg Si/mL)

**SUPPLEMENTAL FIGURE 1.** Schematic of the mouse whole aorta, showing the different segments that were used for biomechanical testing and histochemical staining in Study 2. The whole aorta was excised, cleaned of adherent fat and separated into three segments. The abdominal aorta and the aortic arch were used for histological staining of elastic fibers. The upper segment (namely the thoracic aorta) between the aortic arch and the abdominal aorta up to the renal arteries was used for the determination of mechanical properties and cut into 7 ring samples, each 2 mm in length.


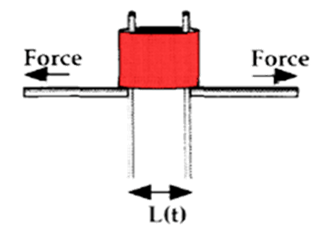

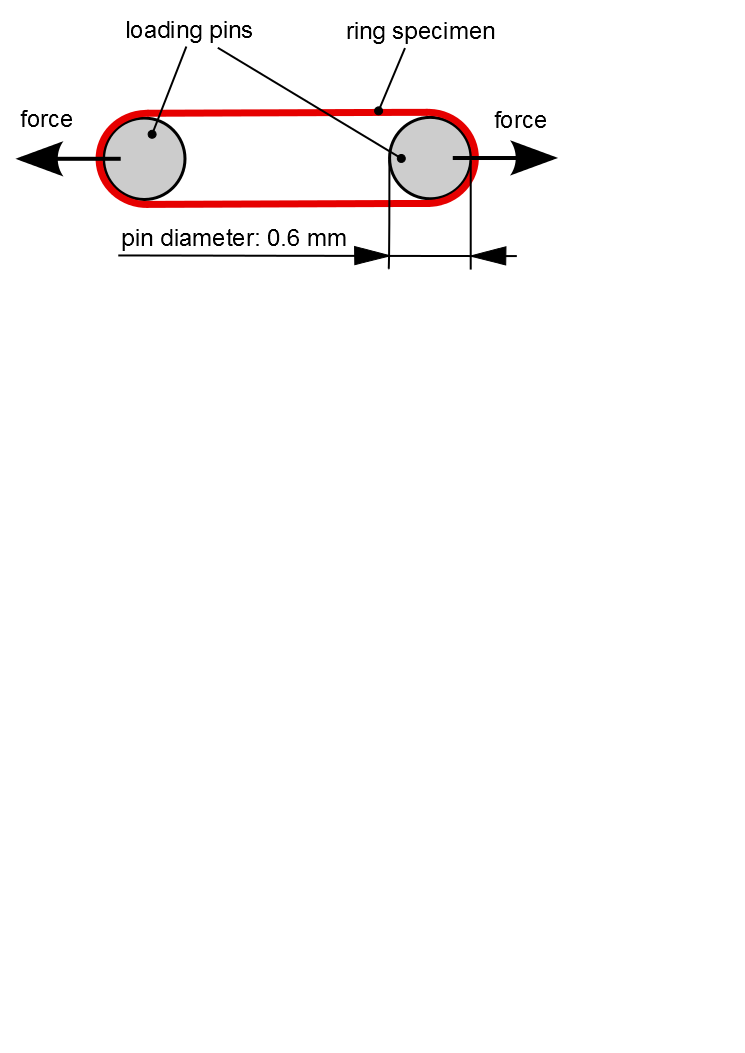


**SUPPLEMENTAL FIGURE 2.** Positioning at the loading pins for biomechanical testing.


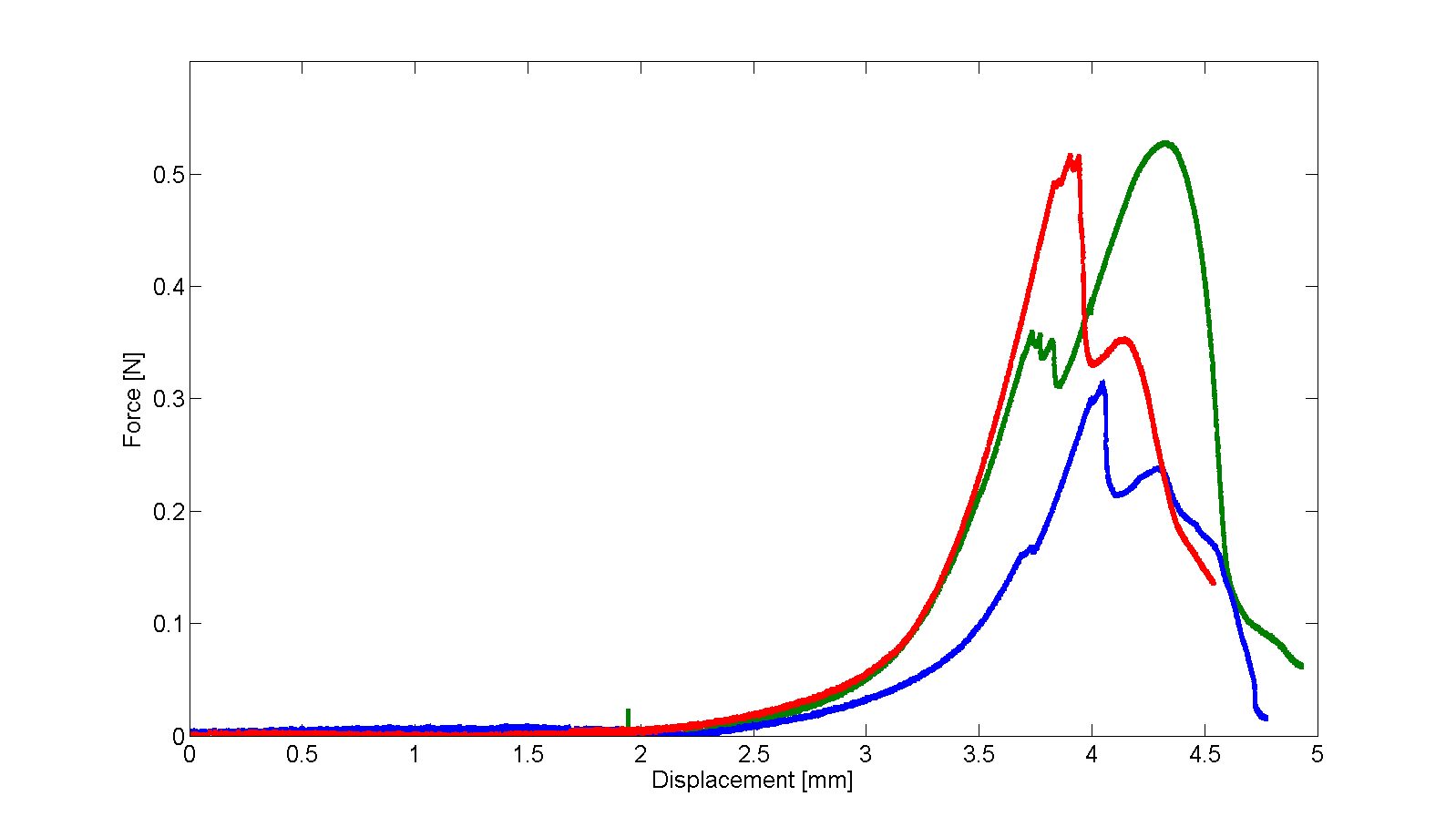


**SUPPLEMENTAL FIGURE 3.** Force-displacement curves from tensile strength measurement of the aortic rings from female Apo-E knockout mice. Three examples are shown; colors do not refer to any particular dietary Si groups.

**SUPPLEMENTAL FIGURE 4**. Wet weights of tissues and organs from the female Apo-E knockout mice in Study 1 (**A**) and Study 2 (**B**) fed a diet high in butter fat and depleted in Si (−Si), compared with mice on the two Si-replete, high-fat diets: Si was replete in the feed (+Si-feed) or in the drinking water (+Si-water). Data are means ± SDs; n = 7-10 for Study 1 and n=10-15 for Study 2. Mice were feed deprived overnight prior to collection of tissues and organs. There was no difference in tissue wet weights between the three dietary Si groups.


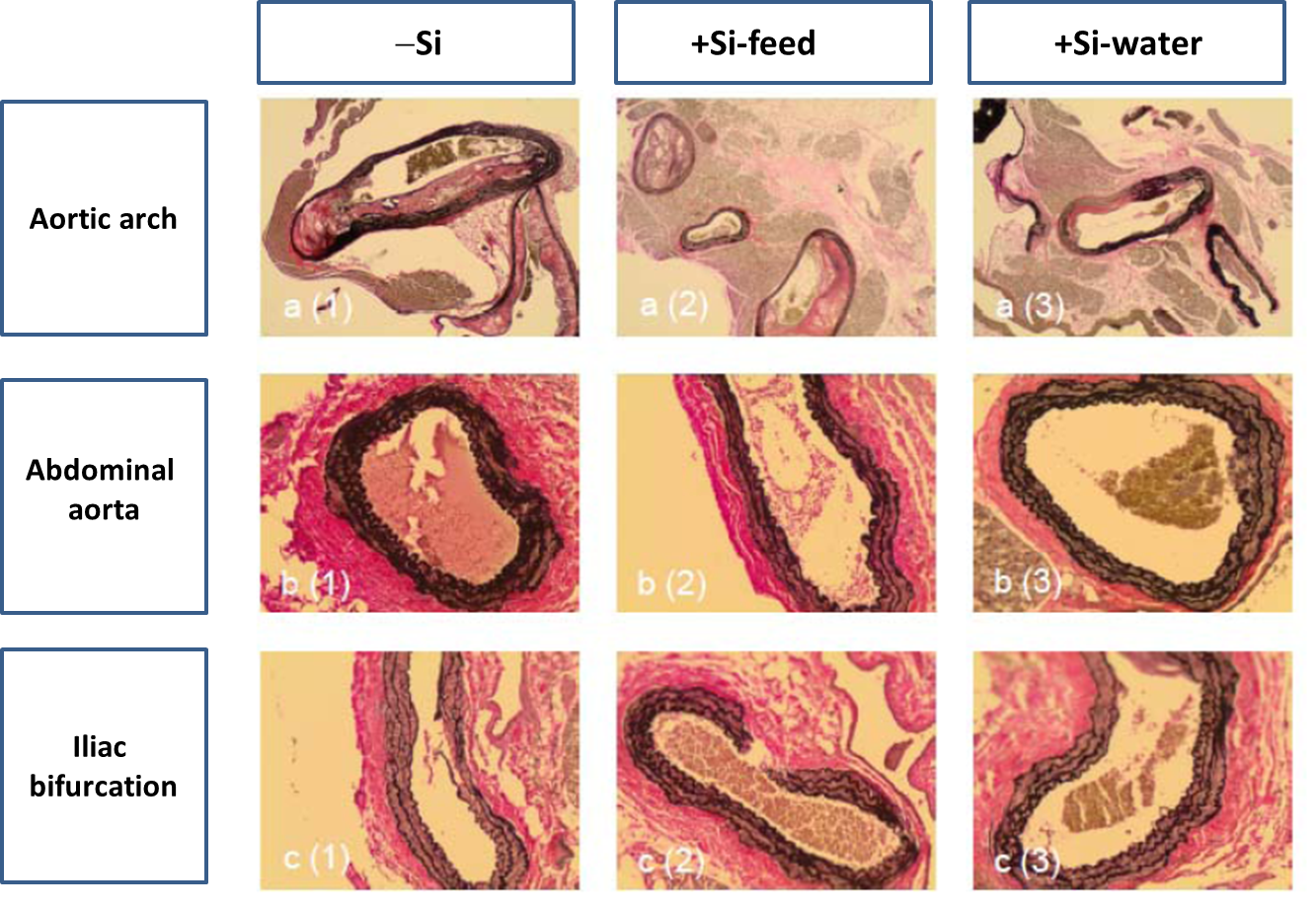


**SUPPLEMENTAL FIGURE 5.** Elastic structure and morphology of the aorta of female Apo-E knockout mice in Study 2, fed a diet high in butter fat and depleted in Si (−Si), compared with mice on the two Si-replete, high-fat diets: Si was replete in the feed (+Si-feed) or in the drinking water (+Si-water). Digital images are cross-sections of the aorta at the aortic arch, abdominal aorta and iliac bifurcation showing the elastic fibers in purple. An example is shown for the three dietary Si groups. Magnification are ×4 (aortic arch) and ×20 (abdominal aorta and iliac bifurcation). The morphological features of the elastic fibers were qualitatively assessed. However, no difference in the structure, size or number of the elastic fibers was observed between three dietary silicon groups.
